# Usability of a point-of-care diagnostic to identify glucose-6-phosphate dehydrogenase deficiency: a multi-country assessment of test label comprehension and results interpretation

**DOI:** 10.1186/s12936-021-03803-1

**Published:** 2021-07-08

**Authors:** Emily Gerth-Guyette, Wondimagegn Adissu, Marcelo Brito, Eduardo Garbin, Marcela Macedo, Abhijit Sharma, Santasabuj Das, Marcus V. G. Lacerda, Dhélio Pereira, Arunansu Talukdar, Daniel Yilma, Sampa Pal, Stephanie Zobrist, Gonzalo J. Domingo

**Affiliations:** 1 PATH, 2201 Westlake Avenue, Suite 200, Seattle, WA 98121 USA; 2 School of Medical Laboratory Sciences, Jimma University, Jimma, Ethiopia; 3 Clinical Trial Unit, Jimma University, Jimma, Ethiopia; 4 Fundação de Medicina Tropical Dr Heitor Vieira Dourado, FMT/HVD), Manaus, Amazonas Brazil; 5 Universidade Do Estado Do Amazonas, Manaus, Amazonas Brazil; 6 Centro de Pesquisa Em Medicina Tropical (CEPEM), Pôrto Velho, Rondônia Brazil; 7 National Institute of Cholera and Enteric Diseases, Indian Council of Medical Research, Kolkata, India; 8 Instituto Leônidas & Maria Deane (ILMD), Fiocruz, Manaus, Amazonas Brazil; 9 Universidade Federal de Rondônia (UNIR), Pôrto Velho, Rondônia Brazil; 10 Department of General Medicine, Medical College, Kolkata, 73 India; 11 Department of Internal Medicine, Jimma University, Jimma, Ethiopia

**Keywords:** G6PD deficiency, Point-of-care diagnostics, Usability, User proficiency, User training, Label comprehension study, Malaria diagnosis

## Abstract

**Background:**

Point-of-care glucose-6-phosphate dehydrogenase (G6PD) testing has the potential to make the use of radical treatment for vivax malaria safer and more effective. Widespread use of G6PD tests as part of malaria case management has been limited, in part due to due concerns regarding product usability, user training, and supervision. This study seeks to assess how well end users can understand the Standard™ G6PD Test (SD Biosensor, Suwon, South Korea) workflow, result output, and label after training. This will ultimately help inform test registration and introduction.

**Methods:**

Potential G6PD test users who provide malaria case management at three sites in Brazil, Ethiopia, and India were trained on the use of the SD Biosensor Standard G6PD Test and assessed based on their ability to understand the test workflow and interpret results. The assessment was done through a questionnaire, designed to assess product usability against key technical product specifications and fulfill regulatory evidence requirements. Any participant who obtained 85% or above correct responses to the questionnaire was considered to adequately comprehend how to use and interpret the test.

**Results:**

Forty-five participants, including malaria microscopists, laboratory staff, nurses, and community health workers took part in the study. Seventy-eight percent of all participants in the study (35/45) obtained passing scores on the assessment with minimal training. Responses to the multiple-choice questions indicate that most participants understood well the test intended use, safety claims, and warnings. The greatest source of error regarding the test was around the correct operating temperature. Most test results were also read and interpreted correctly, with the haemoglobin measurement being a more problematic output to interpret than the G6PD measurement.

**Conclusions:**

These data results show how a standardized tool can be used to assess a user’s ability to run a point-of-care diagnostic and interpret results. When applied to the SD Biosensor Standard G6PD Test, this tool demonstrates that a range of users across multiple contexts can use the test and suggests improvements to the test instructions and training that can improve product usability, increase user comprehension, and ultimately contribute to more widespread effective use of point-of-care G6PD tests.

*Trial registration*: NCT04033640

## Background

Point-of-care (POC) diagnostic testing can be transformative for the management of disease, particularly in resource-constrained settings where laboratory infrastructure is inadequate or overwhelmed by demand. Decentralized testing close to the patient can improve quality of care and better inform treatment options as well as increase the efficiency of health care through more targeted referrals, lower costs, and faster results [1, 2]. This is particularly true in the context of malaria case management, where POC rapid tests have come to replace or supplement the use of microscopic examination of stained blood smears, which is considered the standard of care [3, 4]. While challenges with test accuracy, performance, supply, and operational use persist, these tests have significantly expanded access to malaria diagnosis where it is most needed [5].

POC testing for glucose-6-phosphate dehydrogenase (G6PD) deficiency represents another significant transformation in malaria case management. For patients with *Plasmodium vivax* malaria, widespread screening for G6PD deficiency has the potential to improve health outcomes, align diagnosis and treatment options in remote settings with globally recommended clinical practices, and reduce *P. vivax* transmission through the expanded use of 8-aminoquinolines, including primaquine and tafenoquine (Kozenis®) [6]. G6PD is a critical enzyme in red blood cells. Those with G6PD deficiency express a variant of the enzyme with reduced activity, which can make red blood cells vulnerable to oxidative damage [7]. The approximately 500 million people with this genetic enzymopathy are at a greater risk of experiencing haemolytic anaemia and subsequent clinical complications, particularly when exposed to certain drug triggers, including primaquine [7–10]. Because of the risks associated with G6PD deficiency and primaquine use, the malaria treatment guidelines of the World Health Organization (WHO) recommend that “the G6PD status of patients should be used to guide administration of primaquine” [11]. However, due in part to test availability and operational challenges with G6PD testing at the point of care, national-level policies and practices do not routinely align with these recommendations [12–14].

Until recently, G6PD testing capacity was largely restricted to moderate-to-high throughput tests, only available at national reference laboratories, and some use of near-patient fluorescent spot tests, which still require some laboratory infrastructure and skilled technicians. The recent commercialization of POC G6PD diagnostic tests has begun to address the global gap in test availability [15]. These tests include qualitative rapid diagnostic tests and quantitative or semi-quantitative biosensors that can be readily used at the same levels of the health system where malaria is routinely managed. Qualitative rapid tests may be adequate to differentiate severe deficiency, but they do not reliably differentiate between intermediate and normal levels of G6PD activity; specifically classifying heterozygous females with G6PD normal and deficient allele and intermediate G6PD activity as normal [16].

For this, biosensors show particular promise for their ability to distinguish between the clinically relevant classifications of G6PD status and provide reliable results in both male and female populations [17–19]. Kozenis is indicated for individual with normal G6PD activity (greater than 70%) and, therefore, requires a G6PD test that can differentiate individuals with intermediate activity from those with normal activity [20, 21]. The exclusion of those with intermediate G6PD status along with more severe deficiencies can only be achieved with semi-quantitative and quantitative biosensor tests that can reliably differentiate G6PD deficient, intermediate and normal individuals.

However, challenges persist in the operationalization of biosensor tests. Some of the primary concerns regarding the adoption of G6PD testing at the point of care and the integration of these tests into malaria case management centre around user training and supervision [22]. Although G6PD biosensor diagnostics are portable and rapid, they are moderately more complex than traditional lateral flow rapid tests, as they involve an instrument component as well as a sample preparation step. In addition, enzymatic assays present specific challenges related to maintaining adequate specimen quality, accounting for the instrument’s sensitivity to operating and storage temperatures and ensuring user adherence to test workflows.

Further, effective use of these tests will require a diverse cadre of intended users—some with limited education and training—to not only run the tests but also to interpret the test result outputs. In the case of quantitative or semi-quantitative biosensor tests that provide a numeric result, one or two numbers, potentially with a decimal point, must be compared against predetermined thresholds that classify patients as having either normal, deficient, or intermediate G6PD status. As such, understanding device usability among intended end users is essential to inform appropriate test introduction, uptake, and ultimately, the ability of the diagnostic to improve health outcomes.

Many user challenges and risks can be mitigated in part by effective and clear training, job aids, test labelling, and instructions for use (IFU). In particular, IFUs are a foundational resource in developing a user’s understanding of a test and can thus either help or hinder user proficiency. Best practices for designing IFUs are well documented, and regulatory authorities, including the WHO, have provided considerable guidance on how to evaluate and refine the content of product labels and instructions [23, 24].

In this study, WHO guidance on methods for qualification of usability of POC G6PD tests was applied to understand the usability of the STANDARD G6PD Test (SD Biosensor, Suwon, South Korea). This study sought to explore the following questions: Can the same cadre of health workers who are currently responsible for malaria diagnosis adopt an additional biosensor test? Does the test labelling and IFU support effective operational use of the test? What additional training methods or materials should be developed and adopted to support real-world use of the test?

Accordingly, the goals of this study were (1) to assess the comprehension of the STANDARD G6PD Test packaging and labelling among intended users and (2) to assess users’ ability to interpret the Standard G6PD Test result.

## Methods

**Fig. 1:**
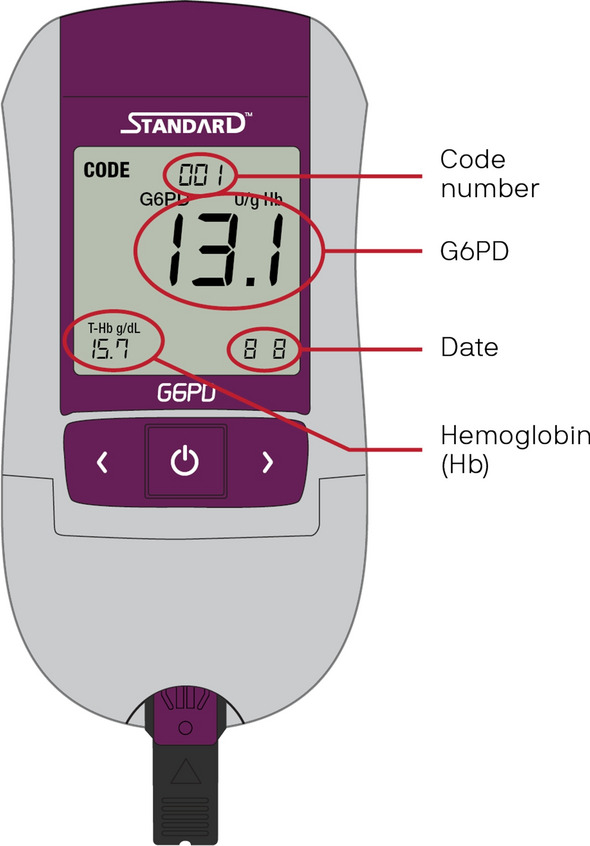
Representation of the SD Biosensor STANDARD G6PD Test and key features on the screen. G6PD: glucose-6-phosphate dehydrogenase; g/dL: grams per decilitre; T-Hb: total haemoglobin

SD Biosensor has developed a quantitative POC diagnostic test for G6PD deficiency—the STANDARD G6PD Test (Fig. 1). This test measures G6PD enzymatic activity and total-haemoglobin concentration in fresh human whole blood specimens (both capillary and venous) based on reflectometry assays and is intended to aid in the identification of people with G6PD deficiency to inform treatment decisions for *P. vivax* at the point of care. The test is registered for use in multiple malaria-endemic countries and has been validated in both malaria-endemic and non-endemic settings [17, 18].

**Fig. 2:**
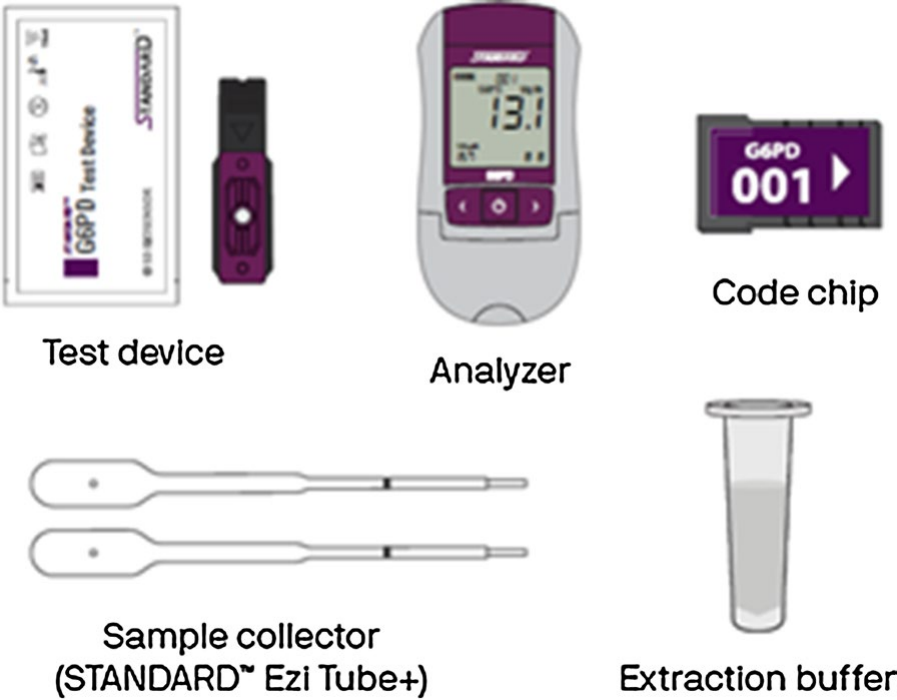
SD Biosensor STANDARD G6PD Test components. Training materials developed by PATH

**Fig. 3:**
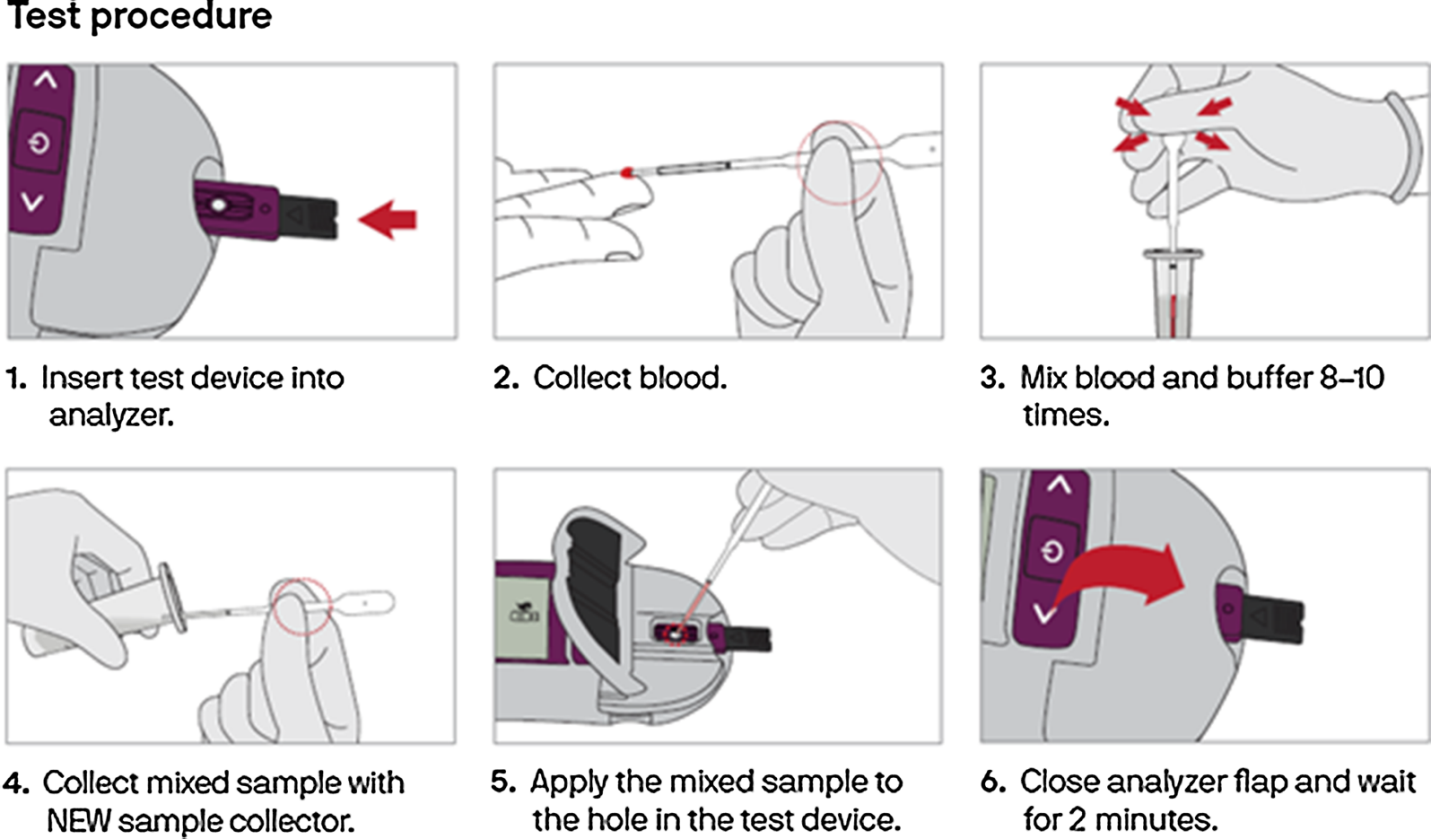
SD Biosensor STANDARD G6PD Test procedure. Training materials developed by PATH

The test includes an instrument, referred to as an analyzer and disposable strips, called test devices. It requires unitized buffer tubes and a unique sample collector called an EziTube+ as well as a lot-specific code chip to calibrate the instrument. The workflow of the test involves collecting a blood sample, using an EziTube+ to mix the blood and buffer, and then transferring the mixed sample to the test device inserted in the analyzer. After 2 min, a quantitative measurement of both G6PD activity and haemoglobin concentration appear on the analyzer screen (Figs. 2, 3).

### Study sites

This usability assessment was conducted at four sites in three countries: two referral hospitals in Brazil, a university in Ethiopia, and a medical college in India. In Brazil, the study was conducted at the Fundação de Medicina Tropical Doutor Heitor Vieira Dourado (FMT/HVD) in Manaus and the Centro de Pesquisa em Medicina Tropical (CEPEM) in Porto Velho. Both sites are clinical reference and research centers for malaria diagnosis and treatment. In Ethiopia, the study was conducted at the Clinical Trial Unit at Jimma University, one of the largest medical schools and tertiary hospitals in Ethiopia, located 350 km southwest of Addis Ababa. The area around Jimma is endemic for malaria, with a large proportion of *P. vivax* malaria, as well as other febrile illnesses. In India, the study was conducted at the Medical College of Kolkata in West Bengal, India. The Medical College of Kolkata and Hospital receive thousands of febrile patients per day, and staff are responsible for supervising the fever clinics in surrounding communities.

### Ethical approvals

All participants in the usability study provided written informed consent to participate. This study received ethical approvals as nested components within three separate diagnostic validation studies. These studies sought primarily to evaluate the performance of the Standard G6PD Test when used by trained health workers in malaria endemic settings (unpublished data). Both the diagnostic accuracy and usability components of the studies received ethical approvals from the following ethical review boards:

- The PATH Research Ethics Committee.
- Brazil: Ethics Committee of FMT/HVD (94833618.0.1001), CEPEM (94833618.0.2001.0011), and the Comissão Nacional de Ética em Pesquisa (CONEP) in Brazil (94,833,618.0.1001.0005).
- Ethiopia: Jimma University Ethics Review Board (INRPGD/416/2018) and the National Research Ethics Review Committee in Ethiopia (NRERC) (3-10/194/2018).
- India: Medical College of Kolkata (MC/Kol/IEC/Spon/113/06-2018) and the National Institute of Cholera and Enteric Diseases Institutional Review Boards (A-1-2/2018/IEC).

### Study design

**Fig. 4:**
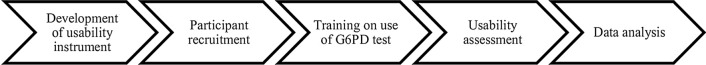
Study approach summary

The study approach followed the process shown in Fig. 4.

#### Development of usability instrument

The usability assessment questionnaire was developed by study staff in line with US Food and Drug Administration guidance on label comprehension studies. The instrument is intended to address key usability risks identified during previous user research and fulfill data requirements from the WHO Prequalification Technical Specifications Series TSS-2 in vitro diagnostics medical devices to identify glucose-6-phosphate dehydrogenase (G6PD) activity document [25, 26].

Thirteen multiple-choice questions with mutually exclusive options and a single correct answer focused on aspects of the product label including key warnings, limitations, and restrictions as well as the proper test procedure. The seven short-answer questions focused on the participant’s ability to read the results screen, record the simulated G6PD and haemoglobin quantitative results, and classify the results as normal, intermediate, deficient, or invalid (Table 1).

**Table 1:** Questionnaire structure and scoring

Question type	Number of questions	Description	Possible points- unweighted analysis	Possible points, analysis weighted by critical errors*
Multiple choice- critical questions	4	Mutually exclusive multiple-choice questions which if missed, would lead to an incorrect test results without the user knowing	13	12
Multiple choice- noncritical questions	9	Mutually exclusive multiple-choice questions which covered background information,		9
Results interpretation	7 (5 valid results, 2 invalid results)	Users were asked to read, record, and interpret predetermined G6PD results based on thresholds provided and told to assume that the results were from female patients	17**	27***
Total possible points			30	48

G6PD: glucose-6-phosphate dehydrogenase

^*^ For each critical error, a weight of 3 points was assigned. For each non critical error, a weight of 1 point was assigned

^**^ For each valid result, three points were possible: one for G6PD result transcription, one for haemoglobin result transcription, and one for result interpretation. For each invalid result, one point was possible

^***^ For each valid result, five points were possible: three for G6PD result transcription, one for haemoglobin result transcription, and one for result interpretation. For each invalid result, one point was possible

#### Participant recruitment and training

First, the study team developed a definition of POC G6PD test end user relevant for each site. Within these definitions, study participants were recruited and selected as part of a convenience sample by the local study team based on their role alignment as representative users of POC G6PD tests, as well as their availability to participate in the study. In Brazil, participants included both health care providers, such as community health workers/field agents, and laboratory technicians who perform malaria rapid diagnostic tests and other malaria testing near the point of care. In Ethiopia, participants included medical technicians who provide laboratory services at public hospitals and health centers around the district. In India, participants included nurses and community health workers who work primarily in the community clinics around the hospital, as well as some hospital laboratory staff.

Health workers who met the inclusion criteria described above were identified by study staff through discussion with managers and supervisors. Once enrolled, participants were trained on use of the STANDARD G6PD Test by members of the study team with extensive experience with G6PD diagnostics generally and the STANDARD G6PD test specifically.

The training took 2 to 3 h and included the following components: (1) a presentation on G6PD deficiency and the implications for malaria treatment, G6PD testing, and the STANDARD G6PD Test, including a short demonstration video; (2) an in-person demonstration of the STANDARD G6PD Test procedure; (3) hands-on practice using the STANDARD G6PD Test and review of the instructions for use (IFU) and manual. The degree of hands-on practice across sites varied based on the amount of time available with both participants and facilitators. The first component of the training was adapted to each site to include locally relevant information and all training materials were reviewed prior to delivery by the local study team for clarity and completeness. The training materials included presentations, a video, and a competency checklist, all available here: https://www.path.org/programs/diagnostics/gorcop/.

Following the training, participants were given the questionnaire to assess their comprehension of the test label and their ability to interpret the test result outputs. The paper-based questionnaire included standardized questions across all three study sites, with the exception that it was delivered in English at the Ethiopia and India sites and translated into Portuguese at the Brazil sites. All study participants were given as much time as needed to complete the questionnaire, which was delivered either immediately after training or a few hours after training was completed.

### Data analysis

Initially, the data was analysed by giving equal weight to each question. Subsequently, the data was analysed to capture whether the question pertained to a critical or noncritical aspect of the test interpretation and workflow. Critical questions were defined as questions, which if answered incorrectly, would likely lead to the user unknowingly obtaining an incorrect result. Noncritical questions were defined as questions that covered background information, would not lead to an incorrect result if missed, or would trigger an invalid or warning message from the instrument if missed. In the results interpretation section, the correct reading and transcription for G6PD was defined as a critical step whereas the haemoglobin transcription and interpretation was defined as noncritical, as in some malaria care scenarios, different users and ancillary job aids may be involved in the interpretation step. For the weighted analysis, the critical questions were given a weight of 3 points as compared to the noncritical questions, which were given a weight of 1 point. Questions left blank were coded as incorrect. Both analyses are presented below.

For both analyses, success criteria was defined as at least 85% correct responses. This equates to a minimum of 26 out of 30 possible points for the full analysis and 41 out of 48 possible points for the analysis that excludes noncritical questions. Any participant meeting this criterion was considered to adequately comprehend the test IFU, labelling, and process for results interpretation. This success criterion is aligned with general regulatory guidance for label comprehension studies and medical symbols as well agreed upon by malaria control programs that may adopt POC G6PD testing in the future.

Analyses included descriptive statistics and a tabular presentation of findings. All data analysis was conducted using Microsoft Excel. The primary endpoints included the percentage of trained health workers who can accurately comprehend key messaging included in the test packaging and labels and accurately interpret the result output and classify results as either normal, invalid, deficient, or intermediate. Data was anonymized by site and then combined as part of a pooled analysis.

## Results

### Study participants

In total, 45 participants were included in the study. In Brazil, 18 health workers were recruited and enrolled from health care facilities in Manaus (n = 9) and Porto Velho (n = 9) between 13 September and 7 October 2019. In Manaus, participants included three quality control malaria microscopists, four malaria field supervisors, and two malaria field workers. In Porto Velho, participants included one malaria microscopist, two quality control malaria microscopists, and six malaria field workers. In Ethiopia, 15 health workers were recruited and enrolled in April 2019. Participants included 11 medical laboratory technicians recruited from health centers and hospitals in and around Jimma and four Jimma University Clinical Trial Unit research staff. In India, data were included from 12 health workers that provide malaria testing and treatment services at surrounding community clinics and in the outpatient ward at the Medical College of Kolkata. Participants included five laboratory technicians, five nursing staff, and two health care coordinators. These participants were recruited, enrolled, and assessed at two different times, nine in September 2019 and an additional three in March 2020. Results are presented below with the countries anonymized as A, B, and C.

### Multiple-choice questions

The responses to the multiple-choice questions indicate that the weakest point of user comprehension was related to the operating temperature in the intended use, with only close to half of all participants identifying the correct operating temperature for the test (Table 2). Other weak points related to the test workflow, with about 20% of participants indicating incorrect responses to questions that covered various steps of the test procedure. The remaining intended use, safety, and warning questions were answered correctly by more than 85% of participants.

**Table 2:** Pooled multiple-choice question results

#	Question	Total (n = 45)
1	The STANDARD G6PD Test can be used to identify people who have G6PD deficiency	41 (91%)
2	What does the STANDARD G6PD Test measure?	39 (87%)
3	What is the operating temperature range for test operation?	23 (51%)
4	The SD Biosensor STANDARD G6PD Test can be used with which types of samples?	38 (84%)
5	How do you use the code chip?	43 (96%)
6	When do you first insert the test strip into the analyzer?	35 (78%)
**7**	**How do you mix the blood sample and the buffer?**	**45 (100%)**
**8**	**After mixing the buffer and the sample, how much of the mixed specimen should be added to the test strip?**	**35 (78%)**
**9**	**After mixing the sample and the buffer, how long should you wait before applying the mixture to the test strip?**	**37 (82%)**
**10**	**How many EZI Tubes do you need to run one sample?**	**36 (80%)**
11	Can you re-use the test strip?	44 (98%)
12	How can you avoid an injury caused by this test?	39 (87%)
13	Which G6PD result are you most likely to see if your patient has very low G6PD activity and is G6PD deficient?	41 (91%)

n: number; G6PD: glucose-6-phosphate dehydrogenase

Critical questions in the weighted analysis are in bold

### Results interpretation

Table 3 presents the results of the short-answer label comprehension questions. Among all participants across all sites, the majority of G6PD activity and haemoglobin measurements were read from the simulated results screen and recorded accurately, apart from four incorrect haemoglobin measurements at site B. The majority of errors resulted from questionnaire responses being left blank by participants.

**Table 3:** Pooled results interpretation results

#	Correct response (G6PD value; Hb value; result interpretation)	Total (n = 45)		Response, if interpretation was missed
		All correct (n, %)	Missed interpretation (n, %)	
14	**13.1 U/g Hb**; 15.7 Hb g/dL; Normal	44 (98%)	1 (2%)	Blank (1)
15	**0.7 U/g Hb**; 11.1 Hb g/dL; Deficient	40 (89%)	5 (11%)	Intermediate (2) Blank (2) Test not working (1)
16	**4.5 U/g Hb**; 13.4 Hb g/dL; Intermediate	37 (82%)	8 (18%)	Blank (2) Deficient (5) Normal (1)
17	NA	45 (100%)	0 (0%)	-
18	**2.0 U/g Hb**; 13.2 Hb g/dL; Deficient	38 (84%)	6 (13%)	Blank (4) Intermediate (2) *one participant misread Hb result
19	E−2	44 (98%)	1 (2%)	Blank (1)
20	**9.2 U/g Hb**; 5.8 Hb g/dL; Normal	37 (82%)	8 (18%)	Blank (4) Intermediate (1) Deficient (3)

G6PD: glucose-6-phosphate dehydrogenase; Hb: haemoglobin; n: number; U/g: units per gram; g/dL: grams per decilitre; NA: not applicable; E: error

Critical transcriptions in the weighted analysis are in bold

All participants correctly classified the invalid results and most participants (91%) correctly classified the normal results. Six participants out of 45 (13%) incorrectly classified an intermediate female result of 4.5 U/g Hb, with five participants indicating a deficient result and one indicating a normal result. Four participants out of 45 (9%) incorrectly classified a normal test result of 9.2 U/g Hb, with three participants indicating a deficient result and one indicating an intermediate result. Other interpretation errors occurred among fewer than three participants per question.

### Summary results by participant

The summary results of the unweighted analysis by participant show that in total, 35 out of 45 participants (78%) met the predetermined acceptance criteria of a minimum of 85% correct responses. There was variation in the passing rate across sites, ranging from 67% of participants meeting the acceptance criteria at country A, 87% at country B, and 83% at country C. The results of the weighted analysis show that 2 additional participants, one at country A and one at country C, met the acceptance criteria, for a total of 37/45 participants or 82% (Table 4).

**Table 4:** Summary results by participants for unweighted and weighted analyses

Country	Participants who met acceptance criteria-unweighted analysis N, (%)	Participants who met acceptance criteria-analysis weighted by critical errors* N, (%)
A	12/18 (67%)	13/18 (72%)
B	13/15 (87%)	13/15 (87%)
C	10/12 (83%)	11/12 (92%)
Total across all countries	35/45 (78%)	37/45 (82%)

## Discussion

Overall, the results of the multiple-choice questions indicated that most participants were able to understand information regarding the test’s intended use, materials, and safety concerns based on the training provided and the resources made available during the assessment.

The most commonly missed questions included (1) *What is the operating temperature range for test operation?* (2) *After mixing the buffer and the sample, how much of the mixed specimen should be added to the test strip?* and (3) *When do you first insert the test strip into the analyzer?* All of which were classified as non-critical.

The most missed question, regarding the operating temperature range, addresses the risk of an operator unknowingly using the test in an environment where the temperature exceeds the acceptable operating range. However, given the test’s wide range of acceptable operating temperatures (15 to 40 °C), the risk of this error occurring is low. It is also likely that the fact that this question was frequently missed was an artefact of the content emphasized during the training: the trainers may not have sufficiently highlighted this aspect of the test.

The second question addresses a critical aspect of the test procedure which, if misunderstood, could potentially impact the result of the test or the ability of the user to obtain a result. However, the instrument design and software include some design features to prevent and lower the risk associated with these types of operator errors. For example, the test includes an error code that would signal to the operator that insufficient sample had been applied to the test strip. The third question addresses a noncritical error, as the instrument will produce an error of the test strip was not fully interested into the analyzer at the beginning of the procedure.

Data from the results interpretation section indicated that participants of all literacy levels can read and record numbers with decimals from the Hindu-Arabic numeral system with very few errors. At site B, where lower-quality printouts of the simulated test readouts were used on the data collection forms, it is possible that the higher rate of transcription errors was a result of this poor print quality. However, misrecording of G6PD test results has been observed and reported both for laboratory-based quantitative tests [27] and the fluorescent spot test [28], so it is important to recognize this risk, but not necessarily as a characteristic specific to the Standard Test. Overall, participants were able to correctly identify the differences between the G6PD activity result and the haemoglobin result and transfer these numbers accurately to paper forms. Most of the missing points in this section were due to blank responses and could be a result of inconsistent instructions on the part of the test proctor or some uncertainly from the participants regarding the application of the thresholds. Results suggest that participants could benefit from clear and reinforced messages around how to apply the thresholds to male and female patient results.

Differences between the unweighted and weighted analysis are minor, with questions weighted by critical or noncritical status resulting in only two additional participants meeting the acceptance criteria. This suggests that in the current data, participant errors are distributed across both critical and noncritical error. Even with a focus on critical errors, most participants (82%) were able to comprehend key aspects of the test procedure and interpret results.

### Mitigating usability risks

These data, reveal what additional tools and materials are needed to support high levels of user effectiveness and satisfaction with POC G6PD tests. Many of the risks associated with the use of the test have been addressed through the test design itself. This includes error messages and other checks embedded in the instrument software as well as a clear IFU document. Residual usability risks as indicated by these data have been subsequently addressed through revisions to the IFU and the development of simplified quick guides with workflow clarifications and key safety information. These quick guides are intended to supplement the traditional IFU, manual, and other test labelling. They rely primarily on images and simple text that can be easily translated to convey key warnings, address common user errors, and reinforce proper test procedure and include a table for results classification as well. These guides are available online through the G6PD Operational Research Community of Practice [29]. They have been translated into multiple languages and the original art files are available for further iteration.

### Informing and planning for broader adoption and use of POC G6PD testing

The availability of POC G6PD tests represents an opportunity for malaria case management to better align with recommended clinical best practices and provide safer and more effective treatments to all malaria patients. As the integration of POC G6PD testing represents a significant shift in malaria case management, malaria control programmes are eager for ways to certify and monitor user competency, and the concept of the usability assessment instrument may serve an important purpose to this end. These instruments must be adaptable to multiple different contexts of malaria service delivery and administered in a decentralized manner.

Since its inception, the data collection instrument highlighted here has been adapted and repurposed to inform training, monitoring, and supervision plans for several pilot studies and other malaria control program activities in Bangladesh, Brazil, and the Mekong region. The results of this study suggest that the instrument will benefit from context-specific adaptations, refining the questions and acceptance criteria to be specific to goals of the supervisors and the health literacy and training level of target users. Careful attention should be paid to the training content, quality, and delivery, as well as the process of proctoring the assessment, as both of these aspects will have a considerable impact on the results. Where possible, trainings should be conducted with small groups of participants, with adequate time for individual hands-on practice and using visual and video training content.

In addition to adequate training and quality assurance for test users and malaria control programs, contextually relevant resources are needed to effectively counsel and educate patients as to the meaning and clinical relevance of their G6PD test results, both for immediate malaria case management and in regard to other risk factors and health implications. Future inquiry should seek to understand what counseling messages regarding G6PD deficiency, a complex genetic condition with multifaceted risk factors and clinical relevance, resonate most with target audiences in malaria-endemic areas.

## Study limitations

There are limitations to this study. Principally, the sampling approach has some limitations in terms of its application to a quantitative methodology and the degree to which the participants included in this study are representative across malaria case management settings. Given the wide range of potential end users of POC G6PD tests globally and across different levels of the health system, the results of this usability assessment may not be generalizable outside of the study sites. The sampling methodology, which involved development of locally-relevant definitions of POC G6PD test end user(s) and then recruiting a convenience sample from health workers at the site that met that definition may further limit generalizability. The modest sample size (n = 45 across 3 countries and 4 sites) was designed in accordance with WHO technical specifications for the quantification of usability but does not support robust sub analyses by site or specific user types, which can be difficult to quantify and compare across contexts. This usability assessment was conducted in view of one specific use case for POC G6PD testing, malaria case management, and with a group of representative end users specific to this context of use.

Even within this use case, participant’s literacy levels and background knowledge varied and either adaptations to the questionnaire based on these differences or data analysis segmented by participants’ literacy and knowledge in the future would be useful. A key limitation to the study is that important differences between the baseline knowledge and medical literacy of participants were not included in the training delivery and data analysis. Additional research, with a larger sample size of each user group, is warranted to better understand the impact of participants baseline knowledge on these findings.

Participants’ results are also highly dependent on the quality of the training provided. Due to differences in language, facilitators, and duration, there was some variation in the training content and delivery across all sites. In Brazil, the training and questionnaire were translated into and delivered in Portuguese whereas in Ethiopia and India the training and questionnaire were delivered in English.

In addition to the differences in training, there were also variations in how the study team proctored the assessment. Emphasis on certain content over other content and logistical issues such as how questions appeared on the test itself might have impacted the results. For example, at some sites, the proctor provided reminders not to leave responses blank. This practice was not consistent across all the study sites and may have impacted study results. Finally, this assessment provides insight only into a user’s comprehension and ability at a single point in time, shortly after the training was conducted. This study does not reflect a user’s ability to interpret results or comprehend the label after weeks or months of cumulative practice with the test. Finally, the scope of the data collection instrument is limited, and was designed as a pragmatic tool to assess key aspects of user comprehension. As POC G6PD testing expands into operational research and routine use, additional user challenges may be highlighted and could be incorporated into further adaptations of this tool.

## Conclusions

These data provide insight into the usability of the SD Biosensor Standard G6PD test as performed by a range of potential end users working in varied roles, across multiple facility types, and from different geographies, to advance the transition toward routine G6PD testing at the point of care. First, these data may serve to inform programs and key decision-makers as to the expected usability of a POC G6PD test in the context of malaria case management. Second, these data indicate what additional user resources are needed to support effective and widespread adoption of decentralized G6PD testing. Finally, these data and the process of data collection offer insights into how these types of user assessments can be adapted; integrated into training, monitoring, and supervision plans; and used to assure user proficiency with decentralized G6PD testing across diverse health workers and levels of the health system.

The use of POC G6PD tests in the context of malaria case management will require laboratory technicians and health care providers working in diverse settings, some with limited education and training, to execute a moderately complex workflow, to interpret results and use them to inform patient care, and to understand the key risks and limitations associated with POC tests. The assessments conducted in Brazil, Ethiopia, and India suggest that this is feasible. All new health technologies, including POC diagnostic tests, will require some training before being rolled out and used across a health system. The results presented here inform where additional training and supervision could be best targeted and how laboratories and clinics can plan for successful introduction of POC G6PD testing.

## Data Availability

The datasets generated and analysed during the current study are available from the corresponding author on reasonable request. The data collection instrument used to generate the data as well as the training tools and materials developed for point-of-care G6PD testing are available here: https://www.path.org/programs/diagnostics/gorcop/.

## Abbreviations

CEPEM: Centro de Pesquisa em Medicina Tropical
CONEP: Comissão Nacional de Ética em Pesquisa
FMT/HVD: Fundação de Medicina Tropical Doutor Heitor Vieira Dourado
G6PD: Glucose-6-phosphate dehydrogenase
IFU: Instructions for use
NRERC: National Research Ethics Review Committee
POC: Point-of-care
WHO: World Health Organization

## References

[1] Kosack CS, Page A-L, Klatser PR (2017). A guide to aid the selection of diagnostic tests. Bull World Health Organ.

[2] Mori M, Ravinetto R, Jacobs J (2011). Quality of medical devices and in vitro diagnostics in resource-limited settings. Trop Med Int Health.

[3] Murray CK, Gasser RA, Magill AJ, Miller RS (2008). Update on rapid diagnostic testing for malaria. Clin Microbiol Rev.

[4] Boyce MR, O’Meara WP (2017). Use of malaria RDTs in various health contexts across sub-Saharan Africa: a systematic review. BMC Public Health.

[5] Wilson ML (2013). Laboratory diagnosis of malaria: conventional and rapid diagnostic methods. Arch Pathol Lab Med.

[6] Baird JK, Valecha N, Duparc S, White NJ, Price RN (2016). Diagnosis and treatment of *Plasmodium vivax* malaria. Am J Trop Med Hyg.

[7] Cappellini MD, Fiorelli G (2008). Glucose-6-phosphate dehydrogenase deficiency. Lancet.

[8] Luzzatto L, Ally M, Notaro R (2020). Glucose-6-phosphate dehydrogenase deficiency. Blood.

[9] Beutler E (1959). The hemolytic effect of primaquine and related compounds: a review. Blood.

[10] Howes RE, Piel FB, Patil AP, Nyangiri OA, Gething PW, Dewi M (2012). G6PD deficiency prevalence and estimates of affected populations in malaria endemic countries: a geostatistical model-based map. PLoS Med..

[11] WHO. Guidelines for the treatment of malaria. 3rd Edn. Geneva, World Health Organization. Accessed November 18, 2019. http://www.who.int/malaria/publications/atoz/9789241549127/en/

[12] Ley B, Luter N, Espino FE, Devine A, Kalnoky M, Lubell Y (2015). The challenges of introducing routine G6PD testing into radical cure: a workshop report. Malar J.

[13] Ley B, Thriemer K, Jaswal J, Poirot E, Alam MS, Phru CS (2017). Barriers to routine G6PD testing prior to treatment with primaquine. Malar J.

[14] Recht J, Ashley EA, White NJ (2018). Use of primaquine and glucose-6-phosphate dehydrogenase deficiency testing: divergent policies and practices in malaria endemic countries. PLoS Negl Trop Dis..

[15] Ley B, Satyagraha AW, Rahmat H (2019). Performance of the Access Bio/CareStart rapid diagnostic test for the detection of glucose-6-phosphate dehydrogenase deficiency: A systematic review and meta-analysis. PLoS Med..

[16] Anderle A, Bancone G, Domingo GJ, Gerth-Guyette E, Pal S, Satyagraha AW (2018). Point-of-care testing for G6PD deficiency: opportunities for screening. Int J Neonatal Screen.

[17] Pal S, Bansil P, Bancone G, Hrutkay S, Kahn M, Gornsawun G (2019). Evaluation of a novel quantitative test for glucose-6-phosphate dehydrogenase deficiency: bringing quantitative testing for glucose-6-phosphate dehydrogenase deficiency closer to the patient. Am J Trop Med Hyg.

[18] Alam MS, Kibria MG, Jahan N, Thriemer K, Richards JS, Domingo GJ (2018). Field evaluation of quantitative point of care diagnostics to measure glucose-6-phosphate dehydrogenase activity. PLoS ONE..

[19] Chu CS, Bancone G, Kelley M, Advani N, Domingo GJ, Cutiongo-de la Paz EM (2020). Optimizing G6PD testing for *Plasmodium vivax* case management: why sex, counseling, and community engagement matter. Wellcome Open Res..

[20] Chu CS, Freedman DO (2019). Tafenoquine and G6PD: a primer for clinicians. J Travel Med..

[21] Commons RJ, McCarthy JS, Price RN (2020). Tafenoquine for the radical cure and prevention of malaria: the importance of testing for G6PD deficiency. Med J Aust.

[22] Kitchakarn S, Lek D, Thol S, Hok C, Saejeng A, Huy R, et al. Implementation of G6PD testing and primaquine for *P. vivax* radical cure: operational perspectives from Thailand and Cambodia. WHO South East Asia J Public Health. 2017;6:60–8. 10.4103/2224-3151.213793 28857064

[23] Jacobs J, Barbé B, Gillet P, Aidoo M, Serra-Casas E, Van Erps J (2014). Harmonization of malaria rapid diagnostic tests: best practices in labelling including instructions for use. Malar J.

[24] Kapp N, Methazia J, Eckersberger E, Griffin R, Bessenaar T (2020). Label comprehension of a combined mifepristone and misoprostol for medical abortion: a pilot study in South Africa. Contraception.

[25] WHO (2016). Technical Specifications Series for submission to WHO prequalification – diagnostic assessment: in vitro diagnostics medical devices to identify Glucose-6-phosphate dehydrogenase (G6PD) activity.

[26] US Food and Drug Administration. Guidance for Industry: Label Comprehension Studies for Nonprescription Drug Products. Published online 2010. https://www.fda.gov/media/75626/download

[27] Tseroni M, Baka A, Kapizioni C (2015). Prevention of malaria resurgence in Greece through the association of mass drug administration (MDA) to immigrants from malaria-endemic regions and standard control measures. PLoS Negl Trop Dis..

[28] Taylor WRJ, Thriemer K, von Seidlein L, Yuentrakul P, Assawariyathipat T, Assefa A (2019). Short-course primaquine for the radical cure of *Plasmodium vivax* malaria: a multicentre, randomised, placebo-controlled non-inferiority trial. Lancet.

[29] G6PD Operational Research Community of Practice. Accessed December 3, 2020. https://www.path.org/programs/diagnostics/gorcop/

